# Immunisation with a Multivalent, Subunit Vaccine Reduces Patent Infection in a Natural Bovine Model of Onchocerciasis during Intense Field Exposure

**DOI:** 10.1371/journal.pntd.0000544

**Published:** 2009-11-10

**Authors:** Benjamin L. Makepeace, Siv Aina Jensen, Sandra J. Laney, Charles K. Nfon, Leo M. Njongmeta, Vincent N. Tanya, Steven A. Williams, Albert E. Bianco, Alexander J. Trees

**Affiliations:** 1 Veterinary Parasitology Group, Liverpool School of Tropical Medicine and Faculty of Veterinary Science, University of Liverpool, Liverpool, United Kingdom; 2 Department of Biological Sciences, Smith College, Northampton, Massachusetts, United States of America; 3 Institut de Recherche Agricole pour le Développement, Wakwa, Adamawa Region, Cameroon; 4 Program in Molecular and Cellular Biology, University of Massachusetts, Amherst, Massachusetts, United States of America; Federal University of Minas Gerais, Brazil

## Abstract

Human onchocerciasis, caused by the filarial nematode *Onchocerca volvulus*, is controlled almost exclusively by the drug ivermectin, which prevents pathology by targeting the microfilariae. However, this reliance on a single control tool has led to interest in vaccination as a potentially complementary strategy. Here, we describe the results of a trial in West Africa to evaluate a multivalent, subunit vaccine for onchocerciasis in the naturally evolved host-parasite relationship of *Onchocerca ochengi* in cattle. Naïve calves, reared in fly-proof accommodation, were immunised with eight recombinant antigens of *O. ochengi*, administered separately with either Freund's adjuvant or alum. The selected antigens were orthologues of *O. volvulus* recombinant proteins that had previously been shown to confer protection against filarial larvae in rodent models and, in some cases, were recognised by serum antibodies from putatively immune humans. The vaccine was highly immunogenic, eliciting a mixed IgG isotype response. Four weeks after the final immunisation, vaccinated and adjuvant-treated control calves were exposed to natural parasite transmission by the blackfly vectors in an area of Cameroon hyperendemic for *O. ochengi*. After 22 months, all the control animals had patent infections (i.e., microfilaridermia), compared with only 58% of vaccinated cattle (*P* = 0.015). This study indicates that vaccination to prevent patent infection may be an achievable goal in onchocerciasis, reducing both the pathology and transmissibility of the infection. The cattle model has also demonstrated its utility for preclinical vaccine discovery, although much research will be required to achieve the requisite target product profile of a clinical candidate.

## Author Summary

River blindness, or onchocerciasis, is caused by a parasitic worm (*Onchocerca volvulus*) that is transmitted by blood-feeding blackflies, which breed in fast-flowing rivers. More than 37 million people are infected and may experience visual impairment and/or severe dermatitis. Control of onchocerciasis is largely dependent on a single drug, ivermectin. Whilst this is extremely effective at killing the worms' offspring (microfilariae) and preventing symptoms, ivermectin does not eliminate the long-lived adult parasites or always stop transmission. Consequently, treatments must be repeated for many years, and drug resistance may be emerging. Against this background, a vaccine against onchocerciasis would provide an important additional tool to sustain effective control. In this study, we evaluated eight worm antigens as vaccine components in cattle, which are often parasitized by *O. ochengi* (the closest relative of *O. volvulus*) in West Africa. Twelve uninfected animals received all eight antigens and were exposed to natural transmission of *O. ochengi* alongside 13 unvaccinated cattle. After almost two years, 92% of vaccinated animals had acquired adult worms, but only 58% were positive for microfilariae; whereas 100% of unvaccinated animals harboured both parasite stages. This suggests that a vaccine against microfilariae to prevent development of disease in humans may be achievable.

## Introduction

Onchocerciasis (‘River Blindness’) is recognised as one of the world's most important neglected tropical diseases [1]. The first-stage larva (microfilaria, Mf) of the nematode *Onchocerca volvulus* causes debilitating lesions of the eyes and skin [2], with >99% of the global burden confined to sub-Saharan Africa [3]. Recent rapid epidemiological mapping of onchocerciasis in central Africa has determined that the prevalence is 37 million [3], more than double that estimated in 1995 [4].

Initially, the main tool for onchocerciasis control was the targeting of riverine breeding sites of the blackfly vector (*Simulium* spp.) with larvicides [5]. However, when the anthelminthic drug ivermectin was donated for human use in 1987, it supplemented vector control in the original Onchocerciasis Control Programme (which ceased operations in 2002) and is now the single tool used for the vast majority of regions covered by the current African Programme for Onchocerciasis Control and the Onchocerciasis Elimination Program for the Americas [6]. Indubitably, ivermectin has been extremely successful in controlling onchocerciasis as a public health problem through annual or semi-annual mass treatments [7]; however, it also has a number of limitations. Firstly, ivermectin is a microfilaricidal drug that is not lethal to the adult worms (i.e., macrofilaricidal) [8]; hence, repeated treatments are required as the adults can persist in the human host for over 10 years [9]. Secondly, ivermectin is contraindicated in areas of central Africa that are hyperendemic for another filarial infection, loiasis, because it can induce a severe post-treatment encephalopathy [10]. Thirdly, ivermectin does not always abrogate transmission, and maintenance of drug distribution for decades is constrained by economic and logistical hurdles, particularly in regions of civil unrest [11]. Finally, there is mounting clinical [12] and molecular [13] evidence that resistance to ivermectin may be emerging in certain foci.

Potential complementary control options for onchocerciasis include a macrofilaricidal drug or a vaccine. The targeting of the *Wolbachia* endosymbionts found within worm tissues with antibiotics has been shown to be macrofilaricidal in *Onchocerca* infections [14],[15] and there has been extensive research into this approach [16]. However, antibiotic chemotherapy is currently not suitable for mass administration since macrofilaricidal activity requires 4–6 weeks of continuous treatment [15]; shorter regimens are not effective [17],[18]. The ambitious objective of vaccine development was the focus of the Edna McConnell Clark Foundation's ‘Oncho Task Force’ network, which facilitated the development of animal models for onchocerciasis, as well as characterisation and production of recombinant antigens and investigations of mammalian immune responses to the parasite [19]. With the recent renewed determination to reduce the global burden of neglected tropical diseases, there has come awareness that even vaccines with only partial efficacy could have a major impact in endemic countries if combined with existing chemotherapeutics [20],[21].

Proof-of-principle for vaccination against onchocerciasis in natural host-parasite relationships has been demonstrated against *O. lienalis* in cattle using sonicated Mf [22] and against *O. ochengi*, also in cattle, using irradiated infective larvae (L_3_) [23]. The latter species is the closest extant relative of *O. volvulus* [24] and is transmitted by the same complex of blackfly vectors (*S. damnosum* sensu lato) in west and central Africa [25]. Moreover, *O. volvulus* and *O. ochengi* exhibit extensive antigenic cross-reactivity, as evidenced by the serological recognition of *O. volvulus* recombinant antigens by cattle infected with *O. ochengi* [26], and can generate cross-protective responses both experimentally [27] and naturally [25]. Therefore, the bovine *O. ochengi* system was utilised to evaluate a recombinant vaccine in a field trial in a hyperendemic area. The vaccine comprised 8 antigens (table 1), originally identified in *O. volvulus*, that were expressed as *O. ochengi* orthologues. These proteins were selected on the basis of extensive research by laboratories within the Oncho Task Force, which used two main criteria: efficacy against filariae in animal models and/or recognition by ‘putatively immune’ sera, obtained from humans who remained apparently uninfected despite intensive natural exposure to *O. volvulus* transmission.

**Table 1:** Characteristics of the antigens.

Designation (GenBank accession no.)	Description	Evidence for protection^b^	Optimal adjuvant	Stage of expression	Percent highly, poorly, and non-immunoresponsive calves^a^	
					IgG1^c^	IgG2^c^
OoALT1 (EU573935)	Secreted larval acidic protein, ‘abundant larval transcript’ [43],[44]	Mouse [44],[45], human [45]	Alum [45]	L_2_, L_3_ [43],[44]	100, 0, 0 (0, 23, 77)	8, 92, 0 (0, 8, 92)
OoB8* (EU573934)	Uncharacterised [45]	Mouse, human [45]	Alum [45]	All [45]	67, 33, 0 (8, 23, 69)	0, 100, 0 (0, 15, 85)
OoRAL2 (EU573933)	Uncharacterised [46]	Mouse^d^, human [46], chimpanzee [47]	Freund's^d^	L_3_, adult [46]	100, 0, 0 (23, 23, 54)	92, 8, 0 (8, 23, 69)
OoTMY1* (EU573931)	Tropomyosin moiety [33]	Human [33], jird [38]	Freund's [38]	All [33]	100, 0, 0 (54, 46, 0)	67, 33, 0 (15, 31, 54)
OoCPI (EU573930)	Cysteine protease inhibitor, ‘onchocystatin’ [48],[49]	Mouse, human [45]	Alum [45]	Egg, L_3_, L_4_, adult [48]	100, 0, 0 (23, 23, 54)	0, 100, 0 (0, 31, 69)
OoB20* (EU573937)	Uncharacterised [50]	Cow, jird [51]	Freund's [51]	Mf, L_2_, L_3_, L_4_ [50]	100, 0, 0 (8, 23, 69)	17, 83, 0 (0, 0, 100)
OoFAR1 (EU573932)	Fatty acid retinoid-binding protein [52]	Jird [53], human [40]	Freund's [53]	All [53]	100, 0, 0 (23, 23, 54)	100, 0, 0 (0, 31, 69)
OoFBA* (EU573936)	Fructose-1,6-bisphosphate aldolase [34]	Mouse [34]	Freund's [34]	All [34]	100, 0, 0 (31, 31, 38)	100, 0, 0 (0, 15, 85)

Notes Mf, microfilaria; L_2–4_, larval developmental stages.

*: These antigens represent truncated polypeptides, not full-length proteins.

a Vaccinated calves (*n* = 12) received all eight antigens in separate inoculations with the respective optimal adjuvant; data in parentheses are comparative values for adjuvant-control animals that received adjuvants only (*n* = 13).

b ‘Mouse’ refers to the *Onchocerca volvulus* L_3_ chamber model; ‘jird’ to the filarial parasite *Acanthocheilonema viteae* in its natural rodent host, *Meriones unguiculatus*; ‘cow’ to *O. lienalis* in its natural host; ‘chimpanzee’ to experimental infection with *O. volvulus*; and ‘human’ to serological recognition by putatively immune individuals from areas endemic for *O. volvulus* infection.

c Highly, poorly and non-immunoresponsive animals exhibited OD_405 nm_>1.0, 0.1–1.0, or <0.1 units (respectively) immediately before natural exposure to infection. Prior correction for non-specific binding was achieved by subtraction of OD_405 nm_ for a pool of negative control sera (obtained from 6 unexposed calves that received neither antigens nor adjuvants).

d D. Abraham, unpublished data.

## Methods

### Animals, field site and ethics

Pregnant cows (*Bos indicus*, Gudali breed) were recruited from the Adamawa Plateau region of Cameroon, and their calves were reared from birth in fly-proof accommodation at the Institut de Recherche Agricole pour le Développement (IRAD), Regional Centre of Wakwa, near Ngaoundéré. The calves were divided into two groups that were matched for age, weight and *O. ochengi* infection status of the dam, as determined by presence or absence of Mf in skin biopsies (table 2). For natural exposure to infection, animals were grazed on pasture bordering the River Vina du Sud for 22 months as previously described [23]. This is a hyperendemic area for *O. ochengi*, where the annual transmission potential has been estimated at 74,000 L_3_ per animal [25]. All procedures performed on animals in Cameroon were equivalent to those authorised by a Home Office Project Licence (Animals [Scientific Procedures] Act 1986) for related work on cattle in the UK. The study was approved by the Ethics Committee of the Regional Centre of Wakwa, IRAD, and authorised by the Regional Programmes Committee of IRAD before experimental work began.

**Table 2:** Experimental animals.

Group (*n ♀, n* ♂)^a^	Median (range) age, weeks^b^	Median (range) weight, kg^b^	Dam status (% infected)^c^
Vaccinated (5, 7)	25 (21–30)	85 (56–120)	75
Adjuvant control (3, 10)	25 (21–30)	96 (64–124)	77

a Both groups originally contained 20 animals, but a total of 15 calves died of causes unrelated to onchocerciasis (predominantly trypanosomiasis) during the first six months of exposure.

b At the time of exposure.

c As determined by microscopic examination of skin biopsies for microfilariae.

### Recombinant antigens

The eight *O. volvulus* antigens selected for the vaccine trial were identified in an *O. ochengi* L_3_-stage Lambda ZAP Express (Stratagene) cDNA library using a standard plaque screening technique (ECL Probe-Amp Kit, Amersham Pharmacia Biotech). Briefly, probes were PCR-labelled with fluorescein using the *O. volvulus* cDNA clones as template. The *O. ochengi* λ-phage plaques were hybridized with the labelled probe to identify the orthologous *O. ochengi* cDNA phage clones, which were then isolated and amplified by PCR. Sequences were verified using a dRhodamine Terminator Cycle Sequencing Kit (Applied Biosystems) on a 310 Genetic Analyzer instrument (Applied Biosystems). The PCR products were sub-cloned (in the appropriate reading frame) into an expression vector incorporating a N-terminal polyhistidine tag (pRSET [Invitrogen] or pJC40 [ATCC]), and the purified plasmids were transformed into BL21(DE3) *Escherichia coli* cells (Invitrogen) for recombinant protein expression. Following analysis by SDS-PAGE, 25 mg of each recombinant fusion protein was purified by metal chelation chromatography (His·Bind Purification Kit, Novagen) according to the manufacturer's instructions. The purified recombinant proteins were dialyzed against 1× phosphate-buffered saline (PBS) and quantified using a bicinchoninic acid protein assay (Pierce).

### Vaccination schedule

Each calf in the vaccinated group received all 8 recombinant antigens as separate injections (a primary immunisation followed by two boosters at 4-week intervals; table 3) in the respective optimal adjuvant (table 1). The proteins were solubilised in sterile PBS, combined with an equal volume of either alum (Imject, Pierce) or Freund's complete adjuvant (Sigma; primary vaccination) followed by Freund's incomplete adjuvant (Sigma; first booster) then PBS only (second booster), and mixed for 10 min until emulsified. To reduce the risk of antigenic competition, each protein was delivered (50% i.m. and 50% s.c.) in a unique muscular site adjacent to a draining lymph node (left or right omotransversarius, triceps, tensor fasciae latae or semitendinosus), and injections in different adjuvants were staggered by two weeks to minimise potential interactions (table 3).

**Table 3:** Schedule of injections.

Injection schedule (weeks)^a^	Dose per antigen (µg)^b^	Vehicle^b^
14	500	FCA
12	500	Alum
10	250	FIA
8	250	Alum
6	250	PBS^c^
4	250	Alum

Notes FCA, Freund's complete adjuvant; FIA, Freund's incomplete adjuvant; PBS, phosphate-buffered saline.

a Preceding natural exposure to infection.

b Adjuvant controls received an equivalent volume of PBS instead of antigen, in combination with vehicle, following an identical schedule to vaccinated animals.

c Final injection in the Freund's series.

### Isotype-specific ELISA

At predetermined intervals, blood was collected by jugular venepuncture and serum was stored at −20°C prior to transport to the UK on refrigerant gel. To reduce non-specific background signals, sera (10% [vol/vol]) were pre-absorbed against *E. coli* extract (2 mg/ml protein, Promega) in blocking solution (20% [vol/vol] soya milk, 10 mM Tris-hydrochloride, pH 8.5; 150 mM sodium chloride, 0.1% [vol/vol] ‘Tween’-20) for 2 h at ambient temperature, followed by overnight incubation at 4°C. Each stage of the assays was optimised independently by checkerboard titration using positive and negative sera pools, obtained from Gudali cattle with patent *O. ochengi* infection (*n* = 9) or 13-week-old Gudali calves reared from birth in fly-proof accommodation (*n* = 6), respectively.

Microtitre plates (MaxiSorp, Nunc) were coated with recombinant antigen in carbonate buffer (15 mM sodium carbonate, 35 mM sodium bicarbonate, pH 9.6) for 24 h (4°C), blocked overnight (4°C) and incubated for 2 h (ambient) with sera diluted in blocking solution. Horseradish peroxidase-conjugated, sheep anti-bovine IgG1 or IgG2 (both obtained from Serotec) was diluted to 0.2 µg/ml in wash buffer (i.e., blocking solution without soya milk) and applied for 2 h (ambient) followed by addition of substrate-chromogen (0.3 mg/ml diammonium 2,2′-azino-bis[3-ethylbenzothiazoline-6-sulphonate] and 0.1% [vol/vol] hydrogen peroxide in 50 mM sodium citrate buffer, pH 4.0). All washes were performed using a SkanWasher-400 automated instrument (Molecular Devices) and OD was measured at 405 nm on an MRX microplate reader (Dynex Technologies). Plates were only accepted if OD_405 nm_ for positive control sera lay within 10% of a predetermined standard, and sample readings were corrected by subtraction of negative control values prior to analysis.

To validate comparisons between IgG1 and IgG2 levels, a commercial bovine immunoglobulin reference serum (Bethyl Laboratories) was used to coat microtitre plates with known concentrations of IgG1 and IgG2 (0.006-12 µg/ml). Over this range, equivalent IgG concentrations produced OD_405 nm_ with a divergence of <25%. Thus, OD_405 nm_ was indicative of the relative concentrations of IgG1 and IgG2 and did not simply reflect differential avidity of the specific conjugates.

### Parasitology

At quarterly intervals from 6 months post-exposure (mpe), animals were assessed by palpation for intradermal nodules (containing adult worms); the positions of which were marked in situ with tattoo ink and recorded on a ‘hide map’. Triplicate skin biopsies were obtained at the same time-points and Mf densities were determined by microscopy as previously described [28]. At the termination of the experiment, palpation for nodules was performed by an individual blinded to the treatment groups. All nodules were removed under local anaesthesia over a period of several weeks (for welfare reasons) and dissected in PBS to release adult male worms from the female mass. The males were counted and their lengths measured, and the female was examined microscopically for developing embryos or Mf in the uteri (gravidity).

### Statistical analysis

All analyses were performed using SPSS software (v. 15.0; SPSS Inc.), and *P*<.05 was the critical threshold unless otherwise specified. For parasitological data, frequencies were compared using relative risk estimates and Fisher's exact tests in the crosstabs procedure, and medians were analysed by Mann-Whitney *U* tests with exact significance. For serological data, animals were categorised as highly, poorly, or non-immunoresponsive according to cut-offs of >1.0, 0.1–1.0, or <0.1 OD_405 nm_ units (respectively) for sera collected immediately before natural exposure to infection. If a treatment group's responses to an antigen were in a single category, further discrimination was achieved using a cut-off at the 50^th^ percentile. To identify potential interactions between antibody responses to the recombinant antigens in individual vaccinated animals, scatter-plots of OD_405 nm_ for antigen-pairs were inspected visually. An apparent association between responsiveness categories was analysed by Fisher's exact test. The medians of total area-under-curve for antibody responses were compared using Mann-Whitney *U* tests with exact significance, and as 16 individual tests were conducted, the Bonferroni adjustment for multiple comparisons was applied.

## Results

### Effect of vaccination on parasitosis

At 22 mpe, the prevalence of dermal Mf in vaccinated animals was significantly lower (by 42%; *P* = .015, Fisher's exact test) than that observed in adjuvant-control animals (table 4). In contrast, vaccination had no significant effect on adult worm burdens, as measured by nodule load, total worm recovery, number of males, or number of gravid females (table 4). Moreover, the length of male worms was not affected significantly by vaccination (data not shown). Despite the reduced prevalence of Mf in vaccinated cattle, median microfilarial density was equivalent to that for adjuvant-control animals (table 4). There was no statistically significant relationship between positive skin biopsies in calves at the termination of the experiment and positive status of dams (table 2) for Mf before calving (relative risk, 0.98; 95% C.I., 0.64–1.52).

**Table 4:** Prevalence and burden of *O. ochengi* in vaccinated and control animals at 22 months post-exposure.

	Vaccinated	Adjuvant control	*P* a
Frequency of nodule-positive animals	11/12	13/13	0.480^b^
Frequency of Mf-positive animals	7/12	13/13	**0.015** b
Median (range) no. of nodules	9.5 (0–19)	14.0 (1–45)	0.263^c^
Median (range) no. of male worms	12.0 (0–26)	13.0 (1–34)	0.716^c^
Median (range) no. of gravid females	3.5 (0–7)	3.0 (1–9)	0.716^c^
Median (range) total worm recovery	21.5 (0–36)	27.0 (2–79)	0.412^c^
Median (range) Mf density per 100 mg skin	14.0 (0.0–2757.4)	17.5 (0.0–317.2)^d^	0.657^c^

a Bold type indicates statistical significance at the *P*<.05 level.

b Fisher's exact test.

c Mann-Whitney *U* test.

d One animal was negative at this time-point but had been positive on prior occasions.

### Antibody responses to recombinant antigens

All vaccinated animals responded with both IgG isotypes to all 8 antigens (defined as an OD_405 nm_ value >0.1 units above the negative control baseline) immediately prior to field exposure (table 1), a time-point that corresponded to 4 weeks after the final immunisations (table 3). For IgG1, all vaccinated animals were highly immunoresponsive (OD_405 nm_>1.0) to all antigens except OoB8, which was strongly recognised by only two-thirds of immunised calves (table 1). Animals with poor IgG1 responses to OoB8 tended to exhibit higher IgG1 levels for OoFBA, but this association was not statistically significant (*P* = .061, Fisher's exact test). In all cases, IgG2 levels were lower than for IgG1, although >90% of vaccinated animals showed strong recognition of OoRAL2, OoFAR1 and OoFBA with this isotype (table 1).

The majority of adjuvant-control animals did not recognise any of the antigens, with the notable exception of IgG1 responses to OoTMY1 (54% high responders, table 1). Strong IgG1 responses to OoFBA were observed in approximately one-third of control animals, whereas high IgG2 levels in this group were restricted to 1–2 animals recognising OoTMY1 and OoRAL2 (table 1).

**Figure 1:**
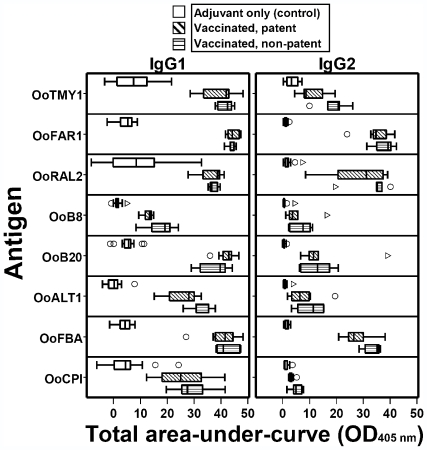
Total area-under-curve (OD_405 nm_) for IgG1 and IgG2 responses to eight *Onchocerca ochengi* recombinant antigens. ‘Vaccinated, patent’ (*n* = 7) or ‘vaccinated, non-patent’ (*n* = 5) refers to presence or absence of dermal microfilariae at 22 months post-exposure, respectively; all adjuvant-control animals had patent infections (*n* = 13). Area-under-curve was calculated from data obtained at 0, 4 and 21 months post-exposure. Box-and-whisker plots display the median line, 25^th^–75^th^ IQR (box), highest and lowest values within 1.5× IQR (whiskers), outliers (○; >1.5–3.0×IQR) and extreme values (▹; >3.0×IQR).

The total area-under-curve was calculated for IgG1 and IgG2 responses at 0, 4 and 21 mpe, and plotted separately for adjuvant-control animals, vaccinated cattle that became patent, and vaccinated animals that were protected from patent infection (figure 1). In general, antibody levels in the vaccinated group peaked at 0–4 mpe; moreover, there was very little (if any) response above baseline to any of the antigens in adjuvant-control cattle following exposure (data not shown). The only marked difference in area-under-curve between patent and non-patent vaccinated animals was observed with the IgG2 response to OoTMY1 (figure 1), with a higher median level exhibited by protected cattle (Mann-Whitney *U* test, *P* = .048). However, this was not statistically significant after Bonferroni adjustment for 16 comparisons (corrected critical *P* = .003).

## Discussion

This study represents the first field trial of a recombinant antigen vaccine against onchocerciasis, and builds upon our preceding evaluation of an irradiated L_3_ vaccine against *O. ochengi* that induced significant protection against natural field challenge [23]. With the recombinant vaccine in the current study, the prevalence of microfilaridermia (i.e., patent infection) was 42% lower than in control animals, whereas the irradiated vaccine induced 67% protection against patency and also significantly reduced the number of gravid female worms and microfilarial density in vaccinated cattle [23]. In addition, immunisations using sonicated *O. lienalis* Mf conferred 97% protection against experimental challenge with homologous Mf in cattle that did not harbour adult parasites [22]. However, a vaccine composed of native parasite material is very unlikely to be produced for human use because of the quantities required, the necessity for cryogenic storage and the infectious risks associated with biological material recovered directly from the host.

The observed protective efficacy against Mf in the current experiment, with no significant effect on the adult stage, is noteworthy. It is possible that the reduction in patent infections in vaccinated animals was secondary to sub-lethal effects on reproduction of the adult parasites [29], although this seems unlikely as there was no apparent abrogation of embryogenesis in female worms. However, since five of the vaccine antigens are expressed in Mf (table 1), the direct targeting of this stage by the immune response is entirely plausible and appears to have occurred without demonstrable vaccine-mediated inhibition of L_3_ development.

A vaccine against Mf might not only be less technically challenging to develop than a prophylactic vaccine directed against L_3_, but could be almost as beneficial to the affected population if pathology was ameliorated and transmission to the vector prevented. Conversely, an anti-Mf vaccine might be associated with a greater risk of inducing immunopathology, particularly as hyper-reactive onchocerciasis (Sowda) is characterised by an aggressive immune response against Mf [30]. This is a hypothetical consideration, but one that would need to be addressed rigorously during clinical testing of any vaccine candidate in onchocerciasis. Whilst Sowda is relatively uncommon in most endemic foci, individuals at increased risk of developing hyper-reactivity to Mf could be identified by genetic screening, since this condition is associated with particular polymorphisms [31],[32]. Moreover, the innate immune responses to *Wolbachia* endobacteria that trigger dermal and ocular pathology in generalised onchocerciasis are a result of the death of Mf in significant numbers [30]; this could be prevented if a vaccine blocked the migration of Mf into the skin and eyes.

The logistic challenges associated with use of a large animal model under tropical field conditions, and the long duration of natural exposure required to test protection (∼2 years), necessitated a multivalent approach in which all vaccinated animals were inoculated with all of the most promising candidate antigens identified in previous studies. Careful design was implemented to diminish competitive inhibition between immune responses by separating the inoculations both anatomically and temporally; consequently, the immunised animals exhibited good immunoresponsiveness to the eight antigens at the levels of IgG1, IgG2, or both isotypes, with little evidence of significant antigen competition. In most cases, specificity of the bovine serological responses was high, although a large proportion of adjuvant-control animals recognised OoTMY1 and OoFBA. Both tropomyosin [33] and fructose-1,6-bisphosphate aldolase [34] are highly conserved proteins, and cross-reactive antibodies could have been generated by co-infections with gastrointestinal nematodes such as *Haemonchus placei*, *Cooperia* spp. and *Strongyloides papillosus*. Indeed, even in housed cattle in the UK, total IgG responses to recombinant *O. volvulus* aldolase were almost indistinguishable between animals experimentally infected with *O. ochengi* and uninfected controls [26].

The disparity between the very high levels of protection afforded by irradiated parasites and some crude antigen extracts, as compared with the recombinant antigens in the current study, could be due to a number of factors. In common with other eukaryotic proteins, the expression of recombinant nematode proteins in *E. coli* can lead to the production of molecules that exhibit aberrant secondary or tertiary structures, or which lack important post-translational modifications. For instance, the *Ancylostoma* secreted proteins have to be expressed in a eukaryotic system (*Pichia pastoris*) in order to attain the conformational epitopes and catalytic activity of the native protein, and these characteristics are critical for the immunogenicity of hookworm vaccines under development [35]. However, all the antigens used in the current field trial had induced significant protection against filarial challenge in other models when expressed as recombinant proteins in *E. coli* (table 1). Perhaps a more relevant limitation to our multivalent approach is the possibility that one or more of the antigens reduced the protective efficacy of the others by the induction of immunoregulatory pathways. Indeed, the *O. volvulus* orthologue of one of the antigens used in the vaccine, OoCPI, can induce hyporesponsiveness in T-cells [36].

There was no compelling association between the serological response to any single antigen and protection against patent infection. However, there was a negative trend (non-significant after Bonferroni correction for multiple comparisons; a highly conservative statistical adjustment [37]) between levels of IgG2 against OoTMY1 and detectable Mf. As the orthologous tropomyosin moiety from *O. volvulus* has been shown to induce protection against *O. lienalis* Mf in a mouse model [38], and anti-tropomyosin antibodies are inversely correlated with Mf density in infected humans [33], this antigen warrants further investigation as a key component of a potential anti-Mf vaccine. This does not necessarily imply that IgG antibodies are the key effectors of vaccine-mediated immunity, but levels of IGg1 and IgG2 were assayed in the current study simply to demonstrate recognition of individual antigens in the immunised animals. Indeed, a previous study of bovine antibody responses to recombinant *O. volvulus* antigens (in Gudali cattle naturally infected with *O. ochengi* at the same field site) reported that neither IgG1 nor IgG2 levels were clearly associated (positively or negatively) with parasite burden [39]. Further insights into the role of OoTMY1 and the other antigens might have been revealed by complementary analyses of IgE levels, lymphoproliferation and eosinophilia [40],[41]. It should be noted that OoTMY1 was delivered in Freund's adjuvant because this had been used in a prior vaccination experiment in jirds, which demonstrated significant protection against a challenge infection with *Acanthocheilonema viteae* [38]. As Freund's adjuvant is not licensed for human use, future trials should consider the inclusion of an alternative adjuvant that could facilitate Th1-like responses, such as CpG oligodeoxynucleotides [42], which may be close to regulatory approval.

In conclusion, we have demonstrated for the first time under field conditions that in a natural host-*Onchocerca* relationship, it is possible to significantly reduce the frequency of infections that attain full patency using a recombinant vaccine. The next phase of vaccine design for onchocerciasis will require the separate evaluation of individual vaccine candidates (particularly tropomyosin) to determine whether the multivalent approach is necessary to achieve protection. The cattle model, although logistically challenging and relatively costly, is far less complex and expensive than are clinical trials in humans. In this field trial and others [14],[17],[23], the *O. ochengi* system has filled a critical niche between laboratory studies in rodent models (that are unnatural hosts of *Onchocerca* parasites) and field evaluation of onchocerciasis control in human populations. Our study opens up the prospect of specifically targeting the Mf stage by vaccination, which in conjunction with currently available chemotherapy, could ensure that the impressive achievements of onchocerciasis control are sustained and extended for the decades to come.
